# Impact of medication adherence to dual antiplatelet therapy on the long-term outcome of drug-eluting or bare-metal stents

**DOI:** 10.1371/journal.pone.0244062

**Published:** 2020-12-16

**Authors:** Jung Min Choi, Seung-Hwa Lee, Mira Kang, Jin-Ho Choi

**Affiliations:** 1 Department of Medical Device Management and Research, Samsung Advanced Institute for Health Sciences & Technology, Sungkyunkwan University, Seoul, Republic of Korea; 2 Department of Medicine, Samsung Medical Center, Sungkyunkwan University School of Medicine, Seoul, Republic of Korea; 3 Healthcare Center, Samsung Medical Center, Sungkyunkwan University School of Medicine, Seoul, Republic of Korea; 4 Samsung Medical Center, Department of Emergency Medicine, Sungkyunkwan University School of Medicine, Seoul, Republic of Korea; Erasmus Medical Center, NETHERLANDS

## Abstract

**Background:**

In percutaneous coronary intervention, drug-eluting stent (DES) showed better clinical outcome compared to bare-metal stent (BMS) but mostly with different DAPT durations.

**Hypothesis:**

The clinical superiority of DES over BMS may depend on the medication adherence to dual antiplatelet therapy (DAPT).

**Methods:**

We retrospectively enrolled all Koreans PCI patients in year 2011 (n = 47,291). Medication adherence to DAPT was assessed by proportion of days covered (PDC) per 6 months. Analysis adjusted with the clinical propensity for receiving DES or BMS and DAPT PDC of the first 6 month was performed. Primary outcome was the 5-year major adverse clinical event (MACE) risk consisting all-cause death, revascularization, shock, or stroke.

**Results:**

Patients with DES (n = 46,356) showed higher PDC (78% versus 60%, p<0.001) and lower MACE risk (39% versus 56%, p<0.001) compared to patients with BMS (n = 935). In the propensity-matched 1,868 patients, MACE risk was lower with DES than BMS (46% versus 54%, HR = 0.80, 95% CI = 0.70–0.91, p<0.001). In both DES and BMS, patients with good medication adherence (PDC ≥80%) showed much lower MACE risk compared to patients with PDC <80% (HR = 0.36, 95% CI = 0.30–0.44; HR = 0.40, 95%CI = 0.33–0.48, p<0.001, all). Patients with DES and PDC <80% showed higher MACE risk compared to BMS with and PDC ≥80% (HR = 1.30, 95%CI = 1.03–1.64, p = 0.027).

**Conclusions:**

Good medication adherence to DAPT in the first 6 month was prerequisite for better clinical outcome in both DES and BMS. DES with poor adherence to DAPT showed worse outcome compared with BMS with good adherence.

## Introduction

Adherence to antiplatelet medications is indispensable for reaching the therapeutic goal after percutaneous coronary intervention (PCI) using drug-eluting stent (DES) or bare metal stent (BMS) [1–4]. Use of DES lowered risk of revascularization as well as risk of stent thrombosis and myocardial infarction compared to BMS [5–11]. In most trials, DAPT duration was typically longer in DES than in BMS. However, in NORSTENT and BASKET-PROVE, two large randomized trials that applied the same DAPT duration in both DES and BMS, there was no difference in the composite outcome of all-cause death and nonfatal myocardial infarction between DES and BMS [8, 12].

This discrepancy among the results of clinical trials suggests that the duration of DAPT may affect the clinical safety and efficacy of DES compared to BMS. Notably, the adherence to DAPT is known to be frequently suboptimal not only in real-world practice but also in clinical trials [13]. Currently, limited data is available for the safety or efficacy of DES compared with BMS with respect to the duration of DAPT.

It is well known that there is a large gap among the real medication adherence measured by electronically traced pills, patient’s self-reported medication, and healthcare professional’s interview [14, 15]. Therefore, we reasoned that medication adherence assessed using pharmaceutical claims may be the best objective substitute for medication duration as a mean of incorporating surveillance of adherence to clinical outcome, and enables utilization of real-world data which should be valuable in observational setting [16]. We investigated the outcome of patients who underwent PCI using DES or BMS with respect to the adherence to DAPT using a nationwide real-world large data.

## Methods

### Study population

The study design was a retrospective all-comer cohort study. Data source was Korean nationwide healthcare database retrieved from the National Healthcare Insurance Service, which is a compulsory healthcare insurance that covers almost all population in Korea. Study cohort consisted of anonymized individual claims of PCI using stent from January 1, 2011 to December 31, 2011. Administrative claims, medical services claims, pharmacy claims, and death records issued from January 1, 2009 through December 31, 2016 were retrieved to assess pre-PCI clinical status and clinical events in the post-PCI follow-up period. Medical services claim data consisted of medical procedure codes and diagnosis codes in the 10^th^ revision of International Statistical Classification of Diseases and Related Health Problems (ICD-10). Data was retrieved on July 1, 2017.

The Samsung Medical Center Institutional Review Board approved this study and determined that this study did not require informed consent given that the analysis used anonymized database and focused on the reporting of aggregated results. This study followed the Strengthening the Reporting of Observational Studies in Epidemiology (STROBE) reporting guidelines and was registered at ClinicalTrials.gov NCT03785509.

### Study definitions and endpoints

The index PCI date was the date of first PCI performed in the selection period from January 1, 2011 to December 31, 2011. Baseline clinical characteristics or medical history prior to the index PCI were defined by respective ICD-10 codes or claims issued in the look-back period between January 1, 2009 and the index PCI date. The procedure or clinical event after PCI were defined by the issued claims and ICD-10 codes in the follow-up period from the index PCI date to the end of study period.

Shock was defined by claims that might be issued in case of cardiogenic shock such as resuscitation, endotracheal intubation and mechanical ventilation, use of hemodynamic support device including intra-aortic balloon counterpulsation or extracorporeal membrane oxygenation. Stroke was defined by corresponding ICD-10 codes accompanied with claims of brain computed tomography or magnetic resonance imaging and hospital admission within 7 days. The overall comorbidity was assessed using Charlson comorbidity index, which categorizes the comorbidity of patient with ICD codes [17]. The details of codes used for clinical status are listed in the S1 File.

Follow-up was determined to be completed if mortality was confirmed or if any medication or administrative claims was issued after index PCI date. No patient was lost to follow-up with respect to death. Follow-up of non-fatal clinical event was completed for 99.2% of the 4^th^ year and 94.8% of the 5^th^ year entries.

DAPT duration was assessed using proportion of days covered (PDC), which is a percentage of days covered with the refill drugs in the number of days the patient is eligible to have the medication. We assumed that PDC ≥ 80%, which is regarded as good medication adherence, as on the clinically effective DAPT. Hence PDC is calculated for a given period, we assessed PDC per 6 month basis and compared PDC of DES and BMS in the first 6 month after PCI. In case of death or follow-up loss, PDC was calculated using the last follow-up date so that PDC was not affected by death or follow-up loss.

The primary outcome was 5-year cumulative risk of a major adverse clinical event (MACE) consisting of all-cause death, revascularization, shock, and stroke. The risks of clinical events were adjusted by the propensity for the use of DES or BMS and the first 6 month PDC of DAPT. Secondary outcomes were 5-year cumulative risk of MACE components and the association of MACE with PDC of DAPT.

### Statistical analysis

Categorical and continuous variables are compared using t-test or chi-square test, appropriately, and shown as mean ± SD or frequency (%), appropriately. The cohort was divided into two groups using DES or BMS. For survival analyses, the potential confounding factors were adjusted by matching patient’s propensity for DES or BMS using a model that included the following parameters; age, gender, prior clinical history including hypertension, diabetes, hyperlipidemia, renal replacement therapy, stroke, malignant neoplasm, prior transfusion, acute myocardial infarction, revascularization, cardiopulmonary resuscitation; initial clinical presentation (angina, non ST-elevation myocardial infarction, ST-elevation myocardial infarction); periprocedural event including transfusion, resuscitation, intubation and mechanical ventilation, use of intra-aortic balloon counterpulsation or extracorporeal membranous oxygenation, gastrointestinal endoscopy, the number of stent used, Charlson’s comorbidity index, and PDC of DAPT at the first 6 month. The discriminative performance of propensity model was acceptable (c-statistics = 0.703, S1 Fig). In the subsequent analyses, propensity score-matched patients that received DES or BMS are classified according to the good (≥ 80%) or poor (< 80%) PDC of DAPT at the first 6 month. Cumulative events of each group are compared using Cox proportional hazard model.

Statistical analysis was performed using SAS version 9.4 (SAS Institute Inc) and R version 3.6 (R Foundation for Statistical Computing). Hazard ratios (HR) for compared outcomes are reported with a 95% confidence interval (CI). Statistical significance was defined by 2-tailed p < 0.05.

## Results

### Characteristics of the study population

**Fig 1:**
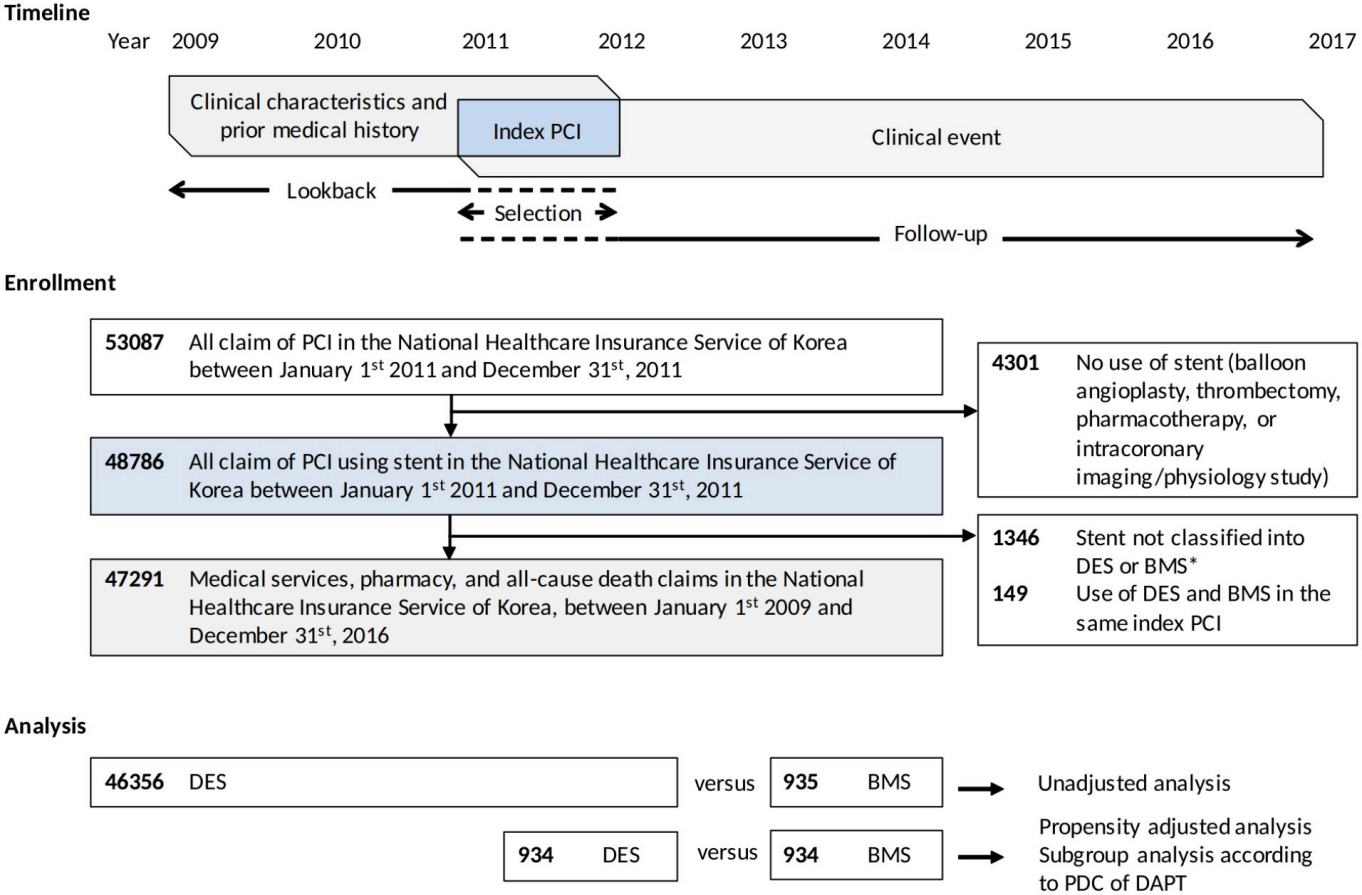
Study flow. *Stent without identified specification.

A total of 53,087 individual PCI procedure was identified in the enrollment period. After exclusion of 5,796 procedures in which both DES and BMS are used simultaneously, without specification of stent, or without use of stent, a total of 47,291 patients with mean age 64.4 ± 11.3 years and 67.7% male gender were enrolled in to the study (Fig 1).

Compared to patients that received BMS, patients that received DES were younger, less often presented with acute coronary syndrome, more often diabetic, more often had prior history of revascularization, and less often had malignant neoplasm (p<0.01, all). Patients that received DES also had lower Charlson’s comorbidity index and were less often in shock (p < 0.001, all). The PDC of DAPT was higher in DES than BMS (p < 0.001) (Table 1).

**Table 1:** Basal clinical and procedural characteristics.

	Unadjusted cohort				Propensity score matched cohort			
	DES	BMS	p-value	SMD	DES	BMS	p-value	SMD
N	46356	935			934	934		
Age (year)	64.4 ± 11.3	67.1 ± 13.1	<0.001	0.219	67.4 ± 11.7	67.1 ± 13.1	0.57	0.030
Male gender	31378 (67.7)	640 (68.4)	0.65	0.016	643 (68.8)	639 (68.4)	0.88	0.009
Hypertension	34506 (74.4)	683 (73.0)	0.36	0.032	684 (73.2)	682 (73.0)	0.96	0.005
Diabetes	11305 (24.4)	198 (21.2)	0.026	0.077	206 (22.1)	198 (21.2)	0.69	0.021
Hyperlipidemia	30753 (66.3)	528 (56.5)	<0.001	0.204	542 (58.0)	528 (56.5)	0.54	0.030
Stroke	2213 (4.8)	55 (5.9)	0.14	0.049	59 (6.3)	55 (5.9)	0.77	0.018
Chronic kidney disease	2516 (5.4)	58 (6.2)	0.34	0.033	61 (6.5)	58 (6.2)	0.85	0.013
Maintenance dialysis	1029 (2.2)	20 (2.1)	0.96	0.006	21 (2.2)	20 (2.1)	1.00	0.007
Malignant neoplasm	2163 (4.7)	136 (14.5)	<0.001	0.34	133 (14.2)	135 (14.5)	0.95	0.006
History of resuscitation	96 (0.2)	3 (0.3)	0.70	0.022	3 (0.3)	3 (0.3)	1.00	<0.001
History of angina	7375 (15.9)	120 (12.8)	0.012	0.088	113 (12.1)	119 (12.7)	0.73	0.019
History of acute myocardial infarction	4057 (8.8)	96 (10.3)	0.12	0.052	105 (11.2)	96 (10.3)	0.55	0.031
History of revascularization	2581 (5.6)	32 (3.4)	0.006	0.104	39 (4.2)	32 (3.4)	0.47	0.039
Percutaneous coronary intervention	2504 (5.4)	28 (3.0)	0.002	0.120	38 (4.1)	28 (3.0)	0.26	0.058
Bypass surgery	92 (0.2)	5 (0.5)	0.06	0.056	1 (0.1)	5 (0.5)	0.22	0.076
Diagnosis								0.076
Angina	28882 (62.3)	430 (46.0)	<0.001	0.332	441 (47.2)	430 (46.0)	0.36	0.066
Non ST-elevation myocardial infarction	6660 (14.4)	190 (20.3)			166 (17.8)	190 (20.3)		
ST-elevation myocardial infarction	10814 (23.3)	315 (33.7)			327 (35.0)	314 (33.6)		
Charlson comorbidity index	0.98 ± 1.49	1.29 ± 1.82	<0.001	0.184	1.28 ± 1.82	1.29 ± 1.82	0.90	0.006
Charlson comorbidity index, category								
0	23277 (50.2)	419 (44.8)	<0.001	0.173	424 (45.4)	419 (44.9)	0.51	0.071
1–2	18017 (38.9)	362 (38.7)			370 (39.6)	361 (38.7)		
3–4	3583 (7.7)	102 (10.9)			83 (8.9)	102 (10.9)		
> = 5	1479 (3.2)	52 (5.6)			57 (6.1)	52 (5.6)		
Stent category								
BMS	-	935 (100)	-	-	-	934 (100)	-	-
DES 1st generation	3615 (7.8)	-			71 (7.6)	-		
DES 1st generation and 2nd generation	659 (1.4)	-			9 (1.0)	-		
DES 2nd generation	42741 (92.2)	-			863 (92.4)	-		
Number of stents	1.12 ± 0.35	1.07 ± 0.27	<0.001	0.171	1.07 ± 0.25	1.07 ± 0.27	0.93	0.004
Periprocedural revascularization	167 (0.4)	5 (0.5)	0.55	0.026	6 (0.6)	5 (0.5)	1.00	0.014
PCI	84 (0.2)	3 (0.3)	0.55	0.028	2 (0.2)	3 (0.3)	1.00	0.021
Bypass surgery	83 (0.2)	2 (0.2)	1.00	0.008	4 (0.4)	2 (0.2)	0.38	0.038
Periprocedural shock	2751 (5.9)	146 (15.6)	<0.001	0.316	148 (15.8)	145 (15.5)	0.90	0.009
Resuscitation and/or hypothermia	1234 (2.7)	76 (8.1)	<0.001	0.244	82 (8.8)	76 (8.1)	0.68	0.023
Endotracheal intubation and/or mechanical ventilation	2316 (5.0)	129 (13.8)	<0.001	0.305	130 (13.9)	128 (13.7)	0.95	0.006
Intraaortic balloon counterpulsation	899 (1.9)	39 (4.2)	<0.001	0.130	40 (4.3)	39 (4.2)	1.00	0.005
Extracorporeal membranous oxygenation	231 (0.5)	14 (1.5)	<0.001	0.101	13 (1.4)	14 (1.5)	1.00	0.009
Periprocedural stroke	1570 (3.4)	47 (5.0)	0.008	0.082	56 (6.0)	47 (5.0)	0.42	0.042
Periprocedural gastrointestinal endoscopy	3265 (7.0)	127 (13.6)	<0.001	0.216	114 (12.2)	126 (13.5)	0.45	0.038
Periprocedural transfusion	3465 (7.5)	164 (17.5)	<0.001	0.308	164 (17.6)	163 (17.5)	1.00	0.003
DAPT PDC at first 6 month (%)	78 ± 35	60 ± 42	<0.001	0.470	59 ± 43	60 ± 41	0.74	0.016

Data are shown with mean (SD) or frequency (%).

### Unadjusted clinical outcome

**Fig 2:**
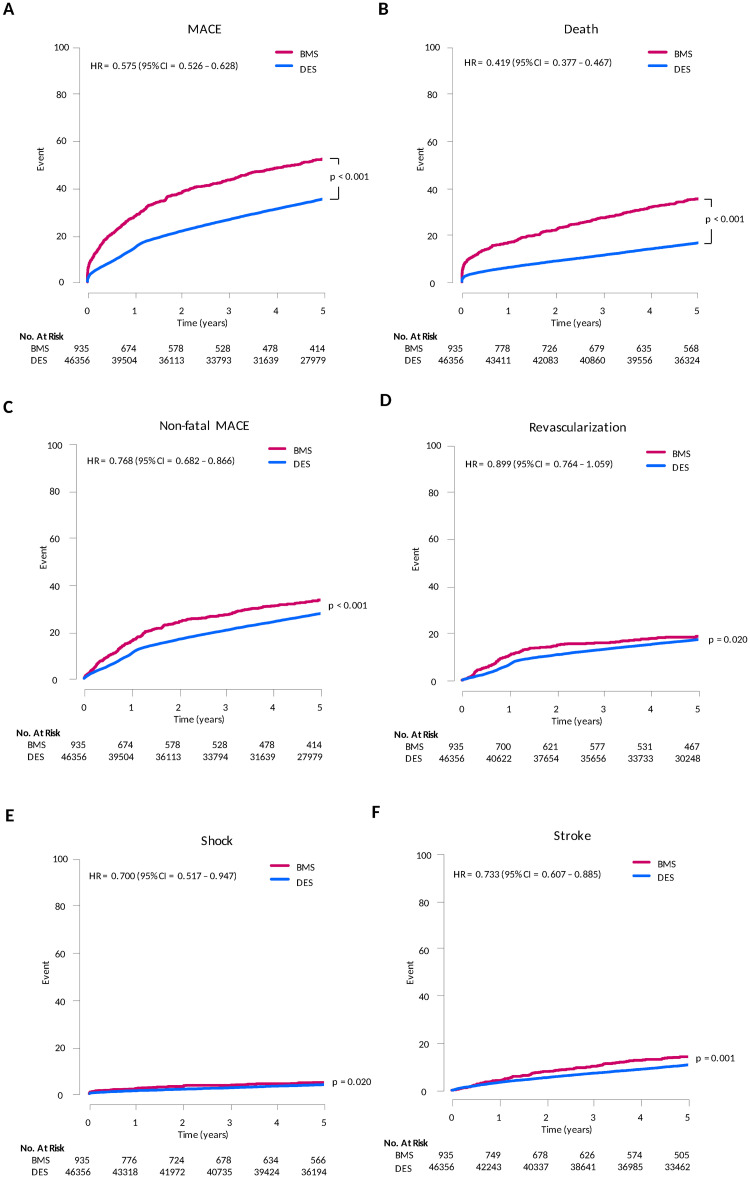
Unadjusted 5-year risk of clinical event. DES showed much lower 5-year cumulative risk of MACE, all-cause death, and non-fatal MACE. However, the risk of revascularization was not different between two groups.

In the analysis of unadjusted whole cohort, DES showed much lower 5-year cumulative risk of MACE compared to BMS (37.8% versus 56.3%, HR = 0.574. 95% CI = 0.526–0.628). DES also showed lower risk of all-cause death (18.3% versus 37.5%, HR = 0.419, 95% CI = 0.377–0.467) and non-fatal MACE (27.4% versus 29.3%, HR = 0.768, 95% CI = 0.682–0.866) (p < 0.001, all). Among components of non-fatal MACE, the risk of shock and stroke were lower in DES compared to BMS (p < 0.05, all), but the risk of revascularization was not different between two groups (Fig 2, S1 Table).

### Clinical outcome adjusted by clinical propensity for the use of DES or BMS

In the survival analysis of propensity matched 934 pairs, the 5-year cumulative risk of MACE DES was lower than BMS (45.9% versus 54.4%, HR = 0.796, 95% CI = 0.700–0.905, p < 0.001). DES also showed lower risk of all-cause death (30.2% versus 37.5%, HR = 0.790, 95% CI = 0.675–0.924, p = 0.003) and non-fatal MACE (24.3% versus 29.3%, HR = 0.774, 95% CI = 0.649–0.923, p = 0.004). The risk of revascularization (12.8% versus 15.6%, HR = 0.780, 95% CI = 0.613–0.993, p = 0.043) was lower in DES, but the risk of shock (3.5% versus 4.6%, HR = 0.754, 95% CI = 0.479–1.187) and stroke (9.6% versus 11.9%, HR = 0.774, 95% CI = 0.586–1.022) were not different between two groups (p > 0.05, all) (S2 Fig).

In survival analyses of clinical subgroups including age, gender, clinical risk factors and comorbidities, prior history of cardiovascular events, prior history of transfusion, initial clinical presentation (angina or myocardial infarction, shock), malignancy, periprocedural transfusion, number of stents, and PDC of DAPT, BMS was not better than DES in terms of MACE and all other clinical events (S3 Fig).

### Survival analysis adjusted by clinical propensity for the use of DES or BMS, and PDC of DAPT at first 6 month

**Fig 3:**
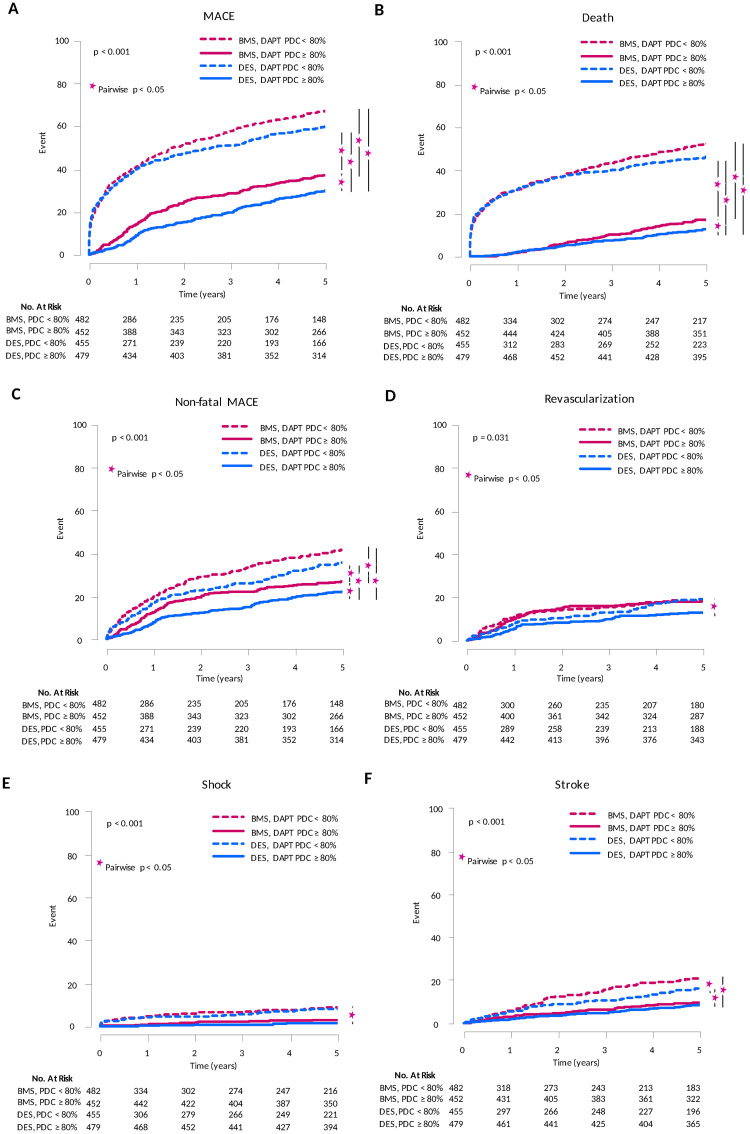
Impact of medication adherence to dual antiplatelet therapy on the propensity score-matched 5-year risk of clinical event. The impact of medication adherence to DAPT on the clinical outcome of propensity score-matched patients was investigate further using PDC ≥ 80% as the threshold of good adherence. Among patients with PDC ≥ 80%, DES showed much lower risk of MACE, all-cause death, and non-fatal MACE. Among patients with PDC < 80%, there was no difference of these risk between DES and BMS. DES with PDC < 80% showed 2-fold higher MACE risk compared to BMS with PDC ≥ 80%.

The impact of medication adherence to DAPT on the clinical outcome of propensity score-matched patients was investigate further using PDC ≥ 80% as the threshold of good adherence. Among patients with PDC ≥ 80%, DES showed much lower risk of MACE, death, and non-fatal MACE (p < 0.05, all). Among patients with PDC < 80%, there was no difference of these risk between DES and BMS (p > 0.05, all). Interestingly, DES with PDC < 80% showed 2-fold higher MACE risk compared to BMS with PDC ≥ 80% (HR = 2.106, 95% CI = 1.744–2.543, p < 0.001) (Fig 3, S2 Table).

Subsequent landmark analysis was performed in order to assess the impact of PDC on the late (> 6 month) clinical events. Among patients with PDC ≥ 80%, there was no difference in the risk of MACE or non-fatal MACE between DES and BMS (p > 0.05, all), but DES showed lower risk of death (HR = 0.647, 95% CI = 0.465–0.900, p = 0.009). Among patients with PDC < 80%, DES showed lower risk of MACE (HR = 0.770, 95% CI = 0.614–0.966, p = 0.024) and death (HR = 0.733, 95% CI = 0.553–0.973, p = 0.031) than BMS, but comparable risk of non-fatal MACE (p > 0.05) (S4 Fig).

## Discussion

In this retrospective analysis of a nationwide real-world data, DES was better than BMS in the composite outcome of death and non-fatal hard clinical events including revascularization, shock, and stroke for up to 5 years in overall. Good medication adherence to DAPT in the first 6 month was strongly associated with lower clinical events in both DES and BMS. These findings were consistent after reflecting baseline clinical profiles and the first 6-month medication adherence to DAPT and also in landmark analysis. Interestingly, DES with poor adherence to DAPT showed worse outcome compared with BMS with good adherence, which emphasize the importance of medication adherence to DAPT in the early period after PCI. To the best of our knowledge, this study is the largest study that compared clinical outcome of DES and BMS with respect to the medication adherence to DAPT.

Our results were derived from a large retrospective registry and may be best appraised by comparing with the results of large scaled clinical studies. Three large meta-analyses showed that the risks of revascularization, stent thrombosis, myocardial infarction, and all-cause death were lower in DES compared to BMS [6, 10, 11]. in these studies, the recommended duration of DAPT was various (1–12 months) and mostly shorter in BMS. However, in NORSTENT and BASKTE-PROVE, DAPT was prescribed for 9 month and 12 month regardless of stent type, respectively, and there was no difference in the composite outcome of all-cause death and nonfatal myocardial infarction between DES and BMS [8, 12]. Our results are in line with these prior results. DES showed lower all-cause death and revascularization compared to BMS in overall. Additionally, good medication adherence in the first 6 month was prerequisite for good clinical outcome not only in DES but also in BMS. Our results suggest that discrepancy among the results of clinical trials might be affected by different DAPT duration or adherence between DES and BMS [5, 8–10, 18].

The optimal duration of DAPT after DES or BMS is still under debate. In DES, longer DAPT beyond the first 6 to 12 months is mostly a trade-off between reducing ischemic event and bleeding event with unclear results on all-cause mortality [19, 20]. In BMS, little has been investigated for the clinical benefit in the use of DAPT for 6 month or longer period. In DAPT trial, continuing DAPT for an additional 18 months compared with placebo among patients with BMS and who tolerated 12 months of DAPT did not result in better clinical outcome, albeit the result was underpowered [21]. In LEADERS FREE trial and a subsequent meta-analysis, polymer-free DES was superior to BMS in patients at high bleeding risk and with 1 month of DAPT [7, 22].

We estimated the DAPT duration based on the PDC ≥ 80%. PDC might be best alternative method that can be applied for large administrative database. EPICOR, a large international prospective observational DAPT duration study, has shown that the use of DAPT decreased gradually but not abruptly across time, and DAPT duration was highly heterogenous among nations [23]. In our study, the cause of DAPT cessation or bleeding was not identified due to limited resource. Further work would be required to discriminate disruption, interruption, and discontinuation of DAPT and their impact on the clinical outcome in large database [2, 24].

Our study did not exclude any high risk patients, which was reflected in apparently high clinical event rate. Interestingly, the 5-year risk of revascularization in our study was 16.7% in DES and 15.6% in BMS, which was numerically comparable to the 6-year risk of revascularization in NORSTENT, 16.5% in DES and 19.8% in BMS [6]. In our study, there was 0.8-fold reduction of clinical composite endpoint in DES compared with BMS in propensity-matched analysis (HR = 0.80, 95% CI = 0.70–0.91), which was numerically similar to those of large meta-analysis (HR = 0.84, 95% CI = 0.78–0.90) [6]. These findings suggest that higher clinical event rate in our study might be driven by underlying higher clinical risk profile rather than coronary lesion or stent characteristics.

### Limitations

The source data was administrative claims which lacked of codes for specific conditions and might not capture patient reported outcome. The data was used as it is without any additional validation study. Although we used multivariable and propensity-modeling analyses, the retrospective observational nature of the data limits mitigation of unmeasured confounding variables, and the identification of causal relationship among choice of stents and clinical outcome. Calculated medication adherence does not represent clinically determined need or cessation of DAPT. The detailed clinical data including severity of disease, laboratory tests, or life styles such as smoking were not available. Myocardial infarction or stent thrombosis were not defined because the results of cardiac biomarkers, electrocardiography, or coronary angiography were not available in administrative database. Instead, we assessed death, revascularization, and shock, which can represent severe complications of myocardial infarction.

## Conclusions

In this real-world cohort data of patients undergoing PCI, DES was better than BMS in the 5-year composite outcome of death and non-fatal hard clinical events including revascularization, shock, and stroke. Good medication adherence to DAPT in the first 6 month was prerequisite for better clinical outcome in both DES and BMS.

## Supporting information

S1 Fig Discriminative performance and balance of propensity score model using clinical parameters for DES or BMS. (PDF)

S2 Fig Propensity score-matched 5-year risk of clinical event. (PDF)

S3 Fig Comparison of DES with BMS in clinical subgroups. (PDF)

S4 Fig Landmark analysis of the impact of medication adherence to dual antiplatelet therapy on the propensity score-matched 5-year risk of clinical event. (PDF)

S1 Table Clinical outcome. (DOCX)

S2 Table Comparison of PDC subgroup in the propensity score-matched 5-year risk of clinical event classified by PDC of DAPT. (DOCX)

S1 File Administrative codes used for retrieve of clinical condition or procedure. (DOCX)

S2 File STROBE statement checklist of items (version 4) for the report of cohort study. (DOCX)
