# Supplementary figures and images for: Impact of medication adherence to dual antiplatelet therapy on the long-term outcome of drug-eluting or bare-metal stents
Source: PLoS One. 2020 Dec 16;15(12):e0244062. doi: 10.1371/journal.pone.0244062 (PMC7743933; doi:10.1371/journal.pone.0244062)

**A**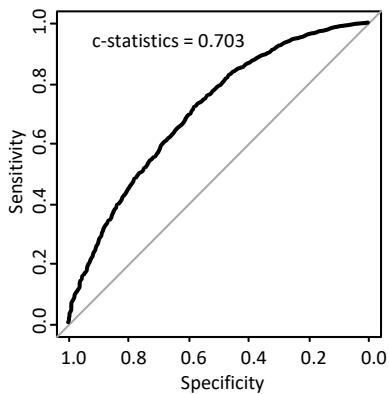**B**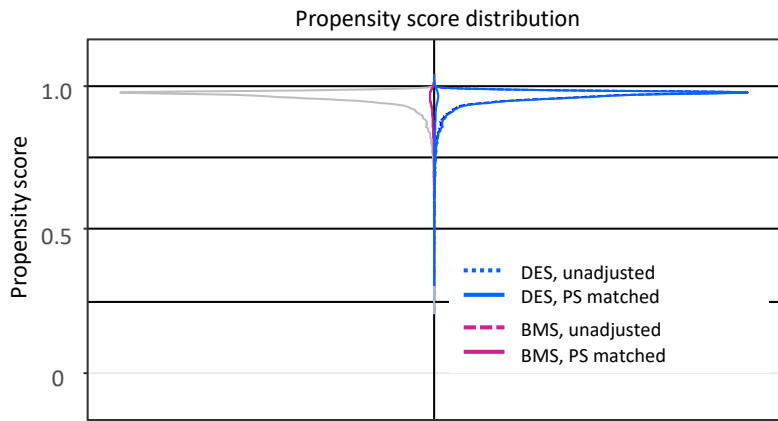

Supplement: S1 Fig — (PDF) [file pone.0244062.s001.pdf]

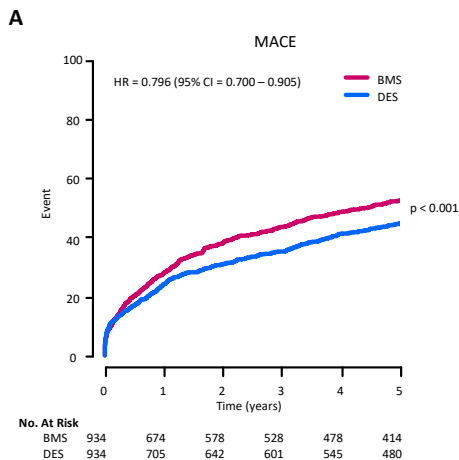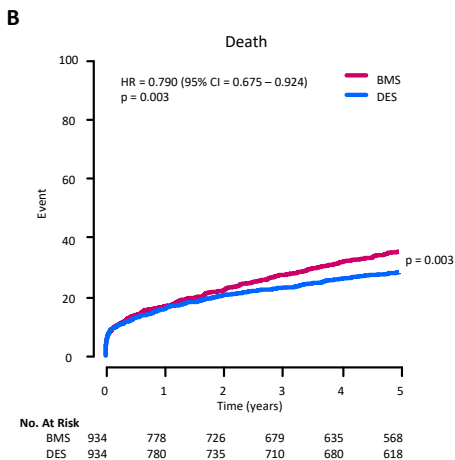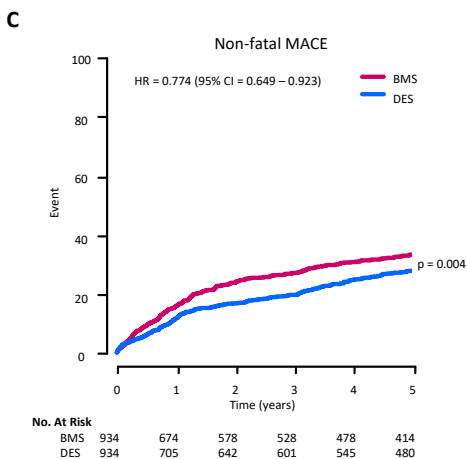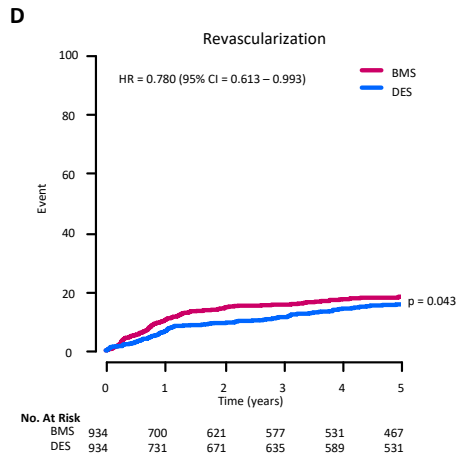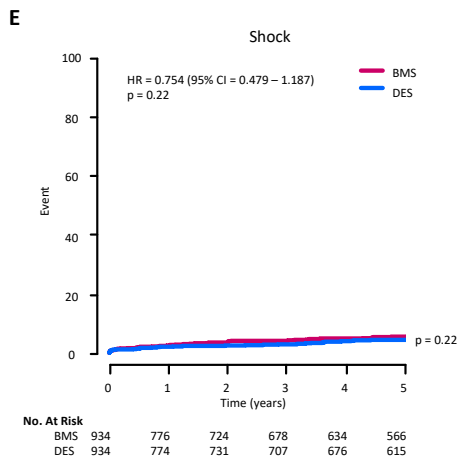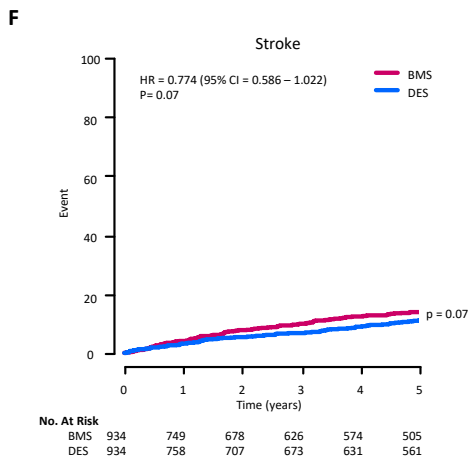

Supplement: S2 Fig — (PDF) [file pone.0244062.s002.pdf]

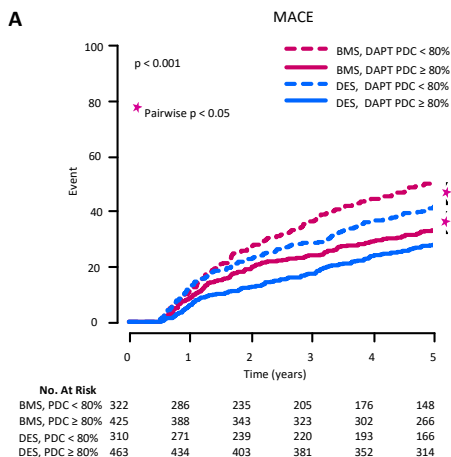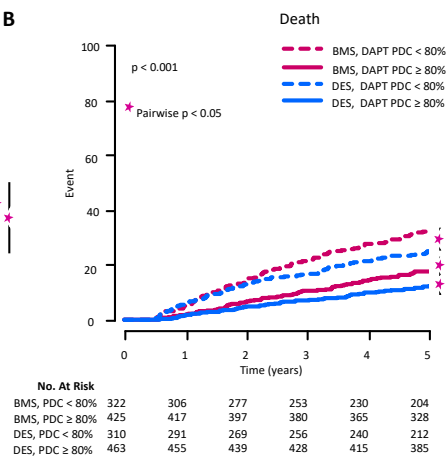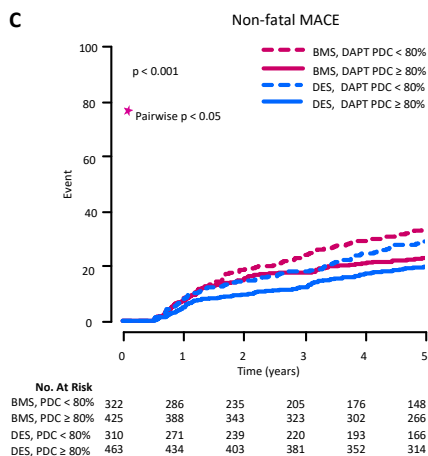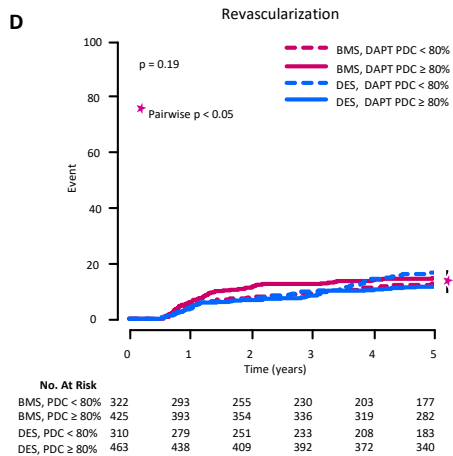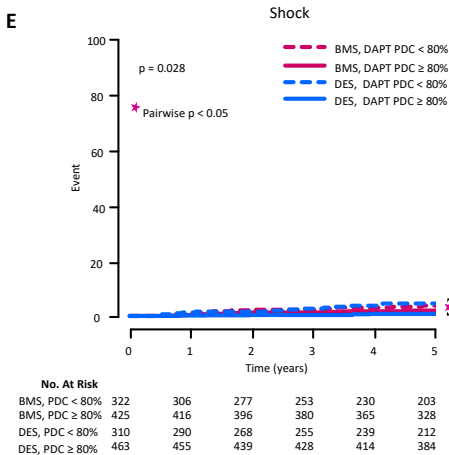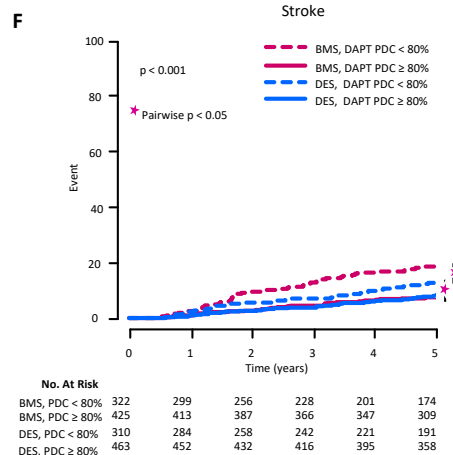

Supplement: S4 Fig — (PDF) [file pone.0244062.s004.pdf]
