# Supplementary material for: Impact of medication adherence to dual antiplatelet therapy on the long-term outcome of drug-eluting or bare-metal stents
Source: PLoS One. 2020 Dec 16;15(12):e0244062. doi: 10.1371/journal.pone.0244062 (PMC7743933; doi:10.1371/journal.pone.0244062)

A Hazard ratio of MACE between stent subtypes

Subgroup		N	HR (95% CI)	p-value	p for interaction
Gender	Male	1282	1.19 (1.02 - 1.40)	0.03	0.25
	Female	586	1.40 (1.12 - 1.73)	<0.01	
Age	≥ 65 years	1142	1.28 (1.10 - 1.49)	<0.01	0.82
	< 65 years	726	1.24 (0.96 - 1.59)	0.10	
Diabetes	Yes	404	1.30 (1.01 - 1.67)	0.04	0.87
	No	1464	1.26 (1.08 - 1.46)	<0.01	
Chronic kidney disease	Yes	119	1.07 (0.70 - 1.63)	0.75	0.38
	No	1749	1.29 (1.12 - 1.47)	<0.01	
Dialysis	Yes	41	0.62 (0.30 - 1.27)	0.19	0.06
	No	1827	1.28 (1.13 - 1.46)	<0.01	
Malignancy	Yes	268	1.43 (1.05 - 1.94)	0.02	0.38
	No	1600	1.23 (1.06 - 1.41)	<0.01	
Stroke	Yes	114	0.93 (0.60 - 1.45)	0.76	0.14
	No	1754	1.29 (1.13 - 1.48)	<0.01	
Prior AMI	Yes	201	1.50 (1.01 - 2.22)	0.05	0.38
	No	1667	1.23 (1.07 - 1.41)	<0.01	
Prior revascularization	Yes	71	1.60 (0.83 - 3.09)	0.16	0.45
	No	1797	1.25 (1.09 - 1.42)	<0.01	
Diagnosis	Angina	871	1.40 (1.15 - 1.71)	<0.01	0.90
	NSTEMI	356	1.40 (1.05 - 1.88)	0.02	
	STEMI	641	1.04 (0.84 - 1.28)	0.73	
Number of stent	1	1750	1.28 (1.12 - 1.47)	<0.01	0.22
	≥ 2	118	0.96 (0.58 - 1.59)	0.87	
Shock	Yes	293	1.04 (0.81 - 1.34)	0.74	0.19
	No	1575	1.33 (1.15 - 1.55)	<0.01	
Periprocedural transfusion	Yes	327	1.21 (0.95 - 1.54)	0.12	0.67
	No	1541	1.30 (1.12 - 1.52)	<0.01	
Charlson's comorbidity index	0	843	1.18 (0.96 - 1.47)	0.12	0.31
	1 - 2	731	1.29 (1.06 - 1.58)	0.01	
	3 - 4	185	1.26 (0.89 - 1.79)	0.20	
	≥ 5	109	1.50 (0.95 - 2.36)	0.08	
DAPT PDC of first 6 month	< 80%	924	1.16 (0.99 - 1.37)	0.07	0.39
	≥ 80%	944	1.34 (1.08 - 1.65)	0.01	

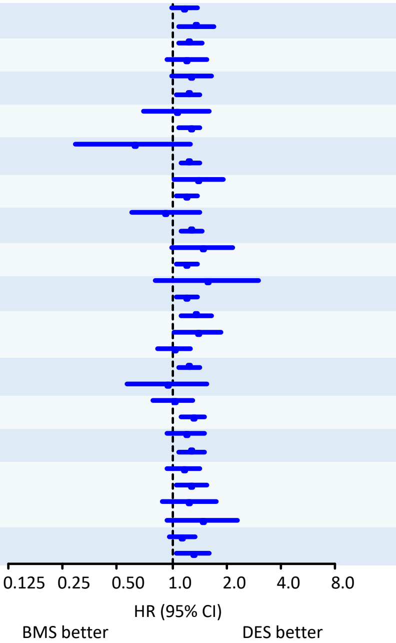

B Hazard ratio of death between stent subtypes

Subgroup		N	HR (95% CI)	p-value	p for interaction
Gender	Male	1282	1.19 (0.98 - 1.46)	0.08	0.25
	Female	586	1.41 (1.09 - 1.82)	0.01	
Age	≥ 65 years	1142	1.30 (1.09 - 1.54)	<0.01	0.82
	< 65 years	726	1.20 (0.81 - 1.78)	0.35	
Diabetes	Yes	404	1.49 (1.10 - 2.02)	0.01	0.87
	No	1464	1.21 (1.00 - 1.45)	0.05	
Chronic kidney disease	Yes	119	1.41 (0.89 - 2.24)	0.15	0.38
	No	1749	1.27 (1.07 - 1.50)	0.01	
Dialysis	Yes	41	0.72 (0.32 - 1.62)	0.43	0.06
	No	1827	1.29 (1.10 - 1.52)	<0.01	
Malignancy	Yes	268	1.48 (1.06 - 2.08)	0.02	0.38
	No	1600	1.22 (1.02 - 1.46)	0.03	
Stroke	Yes	114	0.90 (0.54 - 1.50)	0.69	0.14
	No	1754	1.32 (1.12 - 1.55)	<0.01	
Prior AMI	Yes	201	1.60 (1.01 - 2.53)	0.04	0.38
	No	1667	1.23 (1.04 - 1.45)	0.01	
Prior revascularization	Yes	71	1.65 (0.74 - 3.69)	0.22	0.45
	No	1797	1.25 (1.07 - 1.47)	0.01	
Diagnosis	Angina	871	1.52 (1.17 - 1.98)	<0.01	0.90
	NSTEMI	356	1.49 (1.07 - 2.09)	0.02	
	STEMI	641	0.97 (0.76 - 1.24)	0.82	
Number of stent	1	1750	1.31 (1.11 - 1.54)	<0.01	0.22
	≥ 2	118	0.78 (0.41 - 1.46)	0.43	
Shock	Yes	293	1.00 (0.77 - 1.31)	0.97	0.19
	No	1575	1.43 (1.18 - 1.74)	<0.01	
Periprocedural transfusion	Yes	327	1.25 (0.97 - 1.62)	0.09	0.67
	No	1541	1.33 (1.09 - 1.63)	<0.01	
Charlson's comorbidity index	0	843	1.05 (0.79 - 1.41)	0.72	0.31
	1 - 2	731	1.31 (1.03 - 1.65)	0.03	
	3 - 4	185	1.37 (0.92 - 2.02)	0.12	
	≥ 5	109	1.92 (1.14 - 3.24)	0.01	
DAPT PDC of first 6 month	< 80%	924	1.15 (0.96 - 1.38)	0.14	0.39
	≥ 80%	944	1.46 (1.07 - 1.98)	0.02	

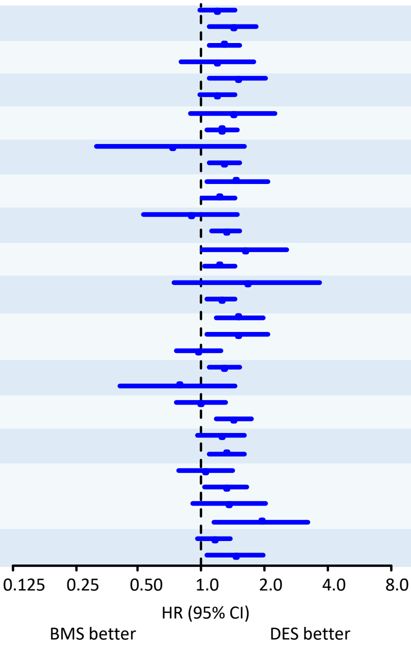

C

Hazard ratio of non-fatal MACE between stent subtypes

Subgroup		N	HR (95% CI)	p-value	p for interaction
Gender	Male	1282	1.15 (0.93 - 1.43)	0.19	0.25
	Female	586	1.64 (1.21 - 2.23)	<0.01	
Age	≥ 65 years	1142	1.34 (1.08 - 1.67)	0.01	0.82
	< 65 years	726	1.22 (0.91 - 1.64)	0.18	
Diabetes	Yes	404	1.15 (0.82 - 1.62)	0.41	0.87
	No	1464	1.36 (1.11 - 1.68)	<0.01	
Chronic kidney disease	Yes	119	1.00 (0.57 - 1.76)	1.00	0.38
	No	1749	1.34 (1.11 - 1.61)	<0.01	
Dialysis	Yes	41	0.64 (0.26 - 1.58)	0.34	0.06
	No	1827	1.32 (1.10 - 1.58)	<0.01	
Malignancy	Yes	268	1.52 (0.94 - 2.46)	0.09	0.38
	No	1600	1.26 (1.04 - 1.52)	0.02	
Stroke	Yes	114	0.88 (0.48 - 1.62)	0.68	0.14
	No	1754	1.34 (1.12 - 1.61)	<0.01	
Prior AMI	Yes	201	1.62 (0.95 - 2.77)	0.07	0.38
	No	1667	1.26 (1.04 - 1.51)	0.02	
Prior revascularization	Yes	71	1.46 (0.63 - 3.36)	0.38	0.45
	No	1797	1.29 (1.08 - 1.54)	0.01	
Diagnosis	Angina	871	1.26 (0.98 - 1.62)	0.07	0.90
	NSTEMI	356	1.43 (0.94 - 2.16)	0.09	
	STEMI	641	1.27 (0.93 - 1.73)	0.13	
Number of stent	1	1750	1.33 (1.11 - 1.60)	<0.01	0.22
	≥ 2	118	0.91 (0.47 - 1.79)	0.79	
Shock	Yes	293	1.34 (0.81 - 2.21)	0.25	0.19
	No	1575	1.28 (1.06 - 1.55)	0.01	
Periprocedural transfusion	Yes	327	1.44 (0.98 - 2.11)	0.06	0.67
	No	1541	1.27 (1.04 - 1.55)	0.02	
Charlson’s comorbidity index	0	843	1.27 (0.96 - 1.67)	0.10	0.31
	1 - 2	731	1.32 (1.00 - 1.74)	0.05	
	3 - 4	185	1.20 (0.74 - 1.96)	0.45	
	≥ 5	109	1.58 (0.79 - 3.16)	0.19	
DAPT PDC of first 6 month	< 80%	924	1.24 (0.98 - 1.58)	0.08	0.39
	≥ 80%	944	1.31 (1.01 - 1.70)	0.04	

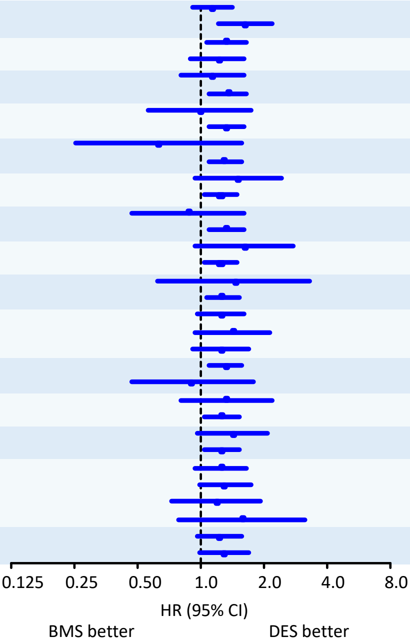

D

Hazard ratio of revascularization between stent subtypes

Subgroup		N	HR (95% CI)	p-value	p for interaction
Gender	Male	1282	1.20 (0.90 - 1.59)	0.21	0.25
	Female	586	1.53 (0.95 - 2.47)	0.08	
Age	≥ 65 years	1142	1.21 (0.88 - 1.68)	0.24	0.82
	< 65 years	726	1.37 (0.95 - 1.98)	0.09	
Diabetes	Yes	404	0.82 (0.49 - 1.37)	0.45	0.87
	No	1464	1.47 (1.11 - 1.94)	0.01	
Chronic kidney disease	Yes	119	1.10 (0.47 - 2.58)	0.83	0.38
	No	1749	1.31 (1.02 - 1.69)	0.04	
Dialysis	Yes	41	0.78 (0.19 - 3.16)	0.72	0.06
	No	1827	1.30 (1.02 - 1.66)	0.04	
Malignancy	Yes	268	0.93 (0.47 - 1.86)	0.84	0.38
	No	1600	1.34 (1.04 - 1.74)	0.03	
Stroke	Yes	114	0.84 (0.31 - 2.25)	0.73	0.14
	No	1754	1.32 (1.03 - 1.70)	0.03	
Prior AMI	Yes	201	1.24 (0.56 - 2.76)	0.60	0.38
	No	1667	1.28 (1.00 - 1.66)	0.05	
Prior revascularization	Yes	71	1.73 (0.58 - 5.15)	0.33	0.45
	No	1797	1.27 (0.99 - 1.62)	0.06	
Diagnosis	Angina	871	1.23 (0.88 - 1.71)	0.22	0.90
	NSTEMI	356	1.71 (0.96 - 3.06)	0.07	
	STEMI	641	1.17 (0.74 - 1.84)	0.50	
Number of stent	1	1750	1.31 (1.02 - 1.69)	0.03	0.22
	≥ 2	118	0.99 (0.37 - 2.65)	0.99	
Shock	Yes	293	1.64 (0.64 - 4.23)	0.31	0.19
	No	1575	1.26 (0.98 - 1.62)	0.07	
Periprocedural transfusion	Yes	327	1.58 (0.81 - 3.09)	0.18	0.67
	No	1541	1.24 (0.96 - 1.61)	0.10	
Charlson’s comorbidity index	0	843	1.45 (1.03 - 2.05)	0.04	0.31
	1 - 2	731	1.32 (0.86 - 2.02)	0.20	
	3 - 4	185	0.95 (0.48 - 1.90)	0.89	
	≥ 5	109	0.69 (0.23 - 2.04)	0.50	
DAPT PDC of first 6 month	< 80%	924	1.03 (0.72 - 1.46)	0.88	0.39
	≥ 80%	944	1.54 (1.10 - 2.14)	0.01	

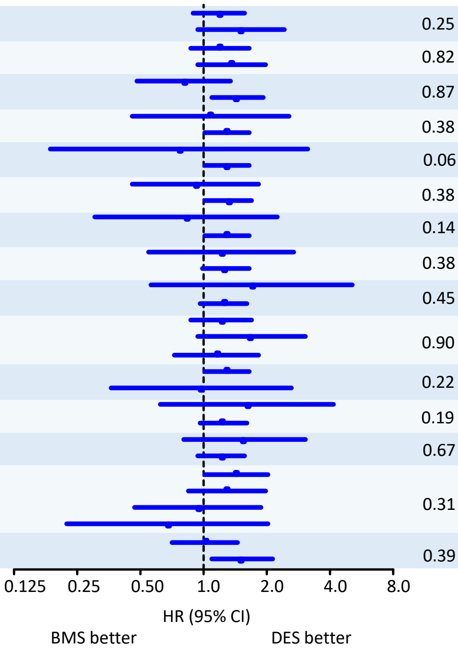

E

Hazard ratio of critically ill cardiovascular status between stent subtypes

Subgroup		N	HR (95% CI)	p-value	p for interaction
Gender	Male	1282	0.93 (0.53 - 1.63)	0.79	0.25
	Female	586	2.59 (1.14 - 5.88)	0.02	
Age	≥ 65 years	1142	1.48 (0.88 - 2.50)	0.14	0.82
	< 65 years	726	0.99 (0.39 - 2.49)	0.98	
Diabetes	Yes	404	1.43 (0.65 - 3.16)	0.37	0.87
	No	1464	1.31 (0.75 - 2.27)	0.34	
Chronic kidney disease	Yes	119	1.80 (0.68 - 4.73)	0.23	0.38
	No	1749	1.28 (0.76 - 2.13)	0.35	
Dialysis	Yes	41	0.63 (0.15 - 2.63)	0.52	0.06
	No	1827	1.46 (0.90 - 2.36)	0.13	
Malignancy	Yes	268	2.52 (0.79 - 8.03)	0.12	0.38
	No	1600	1.15 (0.70 - 1.90)	0.58	
Stroke	Yes	114	2.58 (0.50 - 13.32)	0.26	0.14
	No	1754	1.25 (0.78 – 2.00)	0.36	
Prior AMI	Yes	201	2.39 (0.72 - 7.95)	0.15	0.38
	No	1667	1.21 (0.74 - 1.97)	0.46	
Prior revascularization	Yes	71	1.41 (0.29 - 7.01)	0.67	0.45
	No	1797	1.34 (0.83 - 2.15)	0.23	
Diagnosis	Angina	871	1.06 (0.49 - 2.28)	0.89	0.90
	NSTEMI	356	1.46 (0.63 - 3.37)	0.38	
	STEMI	641	1.45 (0.68 - 3.13)	0.34	
Number of stent	1	1750	1.39 (0.87 - 2.23)	0.17	0.22
	≥ 2	118	0.62 (0.10 - 3.72)	0.60	
Shock	Yes	293	1.33 (0.46 - 3.82)	0.60	0.19
	No	1575	1.33 (0.80 - 2.19)	0.27	
Periprocedural transfusion	Yes	327	1.46 (0.75 - 2.85)	0.27	0.67
	No	1541	1.29 (0.70 - 2.40)	0.41	
Charlson's comorbidity index	0	843	0.33 (0.11 - 1.02)	0.05	0.31
	1 - 2	731	1.47 (0.77 - 2.79)	0.25	
	3 - 4	185	2.72 (0.74 - 10.05)	0.13	
	≥ 5	109	4.76 (1.01 - 22.49)	0.05	
DAPT PDC of first 6 month	< 80%	924	1.01 (0.60 - 1.71)	0.96	0.39
	≥ 80%	944	2.52 (0.97 - 6.56)	0.06	

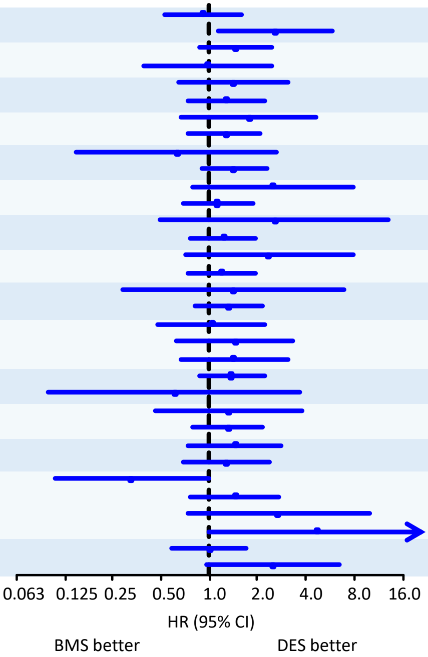

F

Hazard ratio of stroke between stent subtypes

Subgroup		N	HR (95% CI)	p-value	p for interaction
Gender	Male	1282	1.27 (0.89 - 1.82)	0.19	0.25
	Female	586	1.36 (0.87 - 2.11)	0.17	
Age	≥ 65 years	1142	1.48 (1.08 - 2.04)	0.02	0.82
	< 65 years	726	0.92 (0.52 - 1.61)	0.76	
Diabetes	Yes	404	1.46 (0.89 - 2.38)	0.13	0.87
	No	1464	1.25 (0.89 - 1.75)	0.19	
Chronic kidney disease	Yes	119	0.92 (0.37 - 2.28)	0.85	0.38
	No	1749	1.35 (1.01 - 1.81)	0.04	
Dialysis	Yes	41	0.87 (0.17 - 4.31)	0.86	0.06
	No	1827	1.30 (0.98 - 1.73)	0.07	
Malignancy	Yes	268	2.93 (1.14 - 7.57)	0.03	0.38
	No	1600	1.18 (0.88 - 1.59)	0.26	
Stroke	Yes	114	0.80 (0.37 - 1.75)	0.58	0.14
	No	1754	1.40 (1.04 - 1.89)	0.03	
Prior AMI	Yes	201	1.59 (0.72 - 3.49)	0.25	0.38
	No	1667	1.26 (0.94 - 1.70)	0.13	
Prior revascularization	Yes	71	0.90 (0.15 - 5.36)	0.90	0.45
	No	1797	1.30 (0.98 - 1.72)	0.07	
Diagnosis	Angina	871	1.46 (0.99 - 2.17)	0.06	0.90
	NSTEMI	356	0.97 (0.46 - 2.03)	0.93	
	STEMI	641	1.24 (0.78 - 1.96)	0.37	
Number of stent	1	1750	1.32 (0.99 - 1.76)	0.06	0.22
	≥ 2	118	0.98 (0.34 - 2.80)	0.97	
Shock	Yes	293	0.90 (0.38 - 2.13)	0.81	0.19
	No	1575	1.34 (1.00 - 1.80)	0.05	
Periprocedural transfusion	Yes	327	1.17 (0.65 - 2.10)	0.60	0.67
	No	1541	1.35 (0.98 - 1.85)	0.07	
Charlson's comorbidity index	0	843	1.10 (0.67 - 1.80)	0.71	0.31
	1 - 2	731	1.46 (0.97 - 2.21)	0.07	
	3 - 4	185	1.16 (0.59 - 2.29)	0.66	
	≥ 5	109	1.81 (0.55 - 5.93)	0.33	
DAPT PDC of first 6 month	< 80%	924	1.48 (1.03 - 2.14)	0.04	0.39
	≥ 80%	944	1.02 (0.66 - 1.57)	0.93	

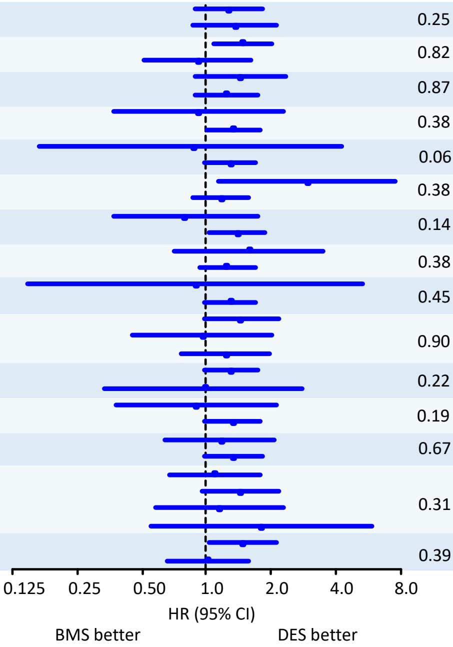

Supplement: S3 Fig — (PDF) [file pone.0244062.s003.pdf]
