# Supplementary material for: Impact of medication adherence to dual antiplatelet therapy on the long-term outcome of drug-eluting or bare-metal stents
Source: PLoS One. 2020 Dec 16;15(12):e0244062. doi: 10.1371/journal.pone.0244062 (PMC7743933; doi:10.1371/journal.pone.0244062)
Supplement: S1 Table — (DOCX) [file pone.0244062.s005.docx]

|  | **Unadjusted cohort** | | |  | **Propensity score matched cohort** | | |
| --- | --- | --- | --- | --- | --- | --- | --- |
|  | **DES** | **BMS** | **p-value** |  | **DES** | **BMS** | **p-value** |
| N | 46356 | 935 |  |  | 934 | 934 |  |
| MACE | 17521 ( 37.8) | 509 ( 54.4) | <0.001 |  | 429 ( 45.9) | 508 ( 54.4) | <0.001 |
| Death | 8473 ( 18.3) | 351 ( 37.5) | <0.001 |  | 282 ( 30.2) | 350 ( 37.5) | 0.001 |
| Non-fatal MACE | 12702 ( 27.4) | 274 ( 29.3) | 0.21 |  | 227 ( 24.3) | 274 ( 29.3) | 0.016 |
| Revascularization | 7762 ( 16.7) | 146 ( 15.6) | 0.38 |  | 120 ( 12.8) | 146 ( 15.6) | 0.1 |
| Stroke | 4831 ( 10.4) | 111 ( 11.9) | 0.17 |  | 90 ( 9.6) | 111 ( 11.9) | 0.14 |
| Shock | 1739 ( 3.8) | 43 ( 4.6) | 0.21 |  | 33 ( 3.5) | 43 ( 4.6) | 0.29 |
