# Supplementary material for: Impact of medication adherence to dual antiplatelet therapy on the long-term outcome of drug-eluting or bare-metal stents
Source: PLoS One. 2020 Dec 16;15(12):e0244062. doi: 10.1371/journal.pone.0244062 (PMC7743933; doi:10.1371/journal.pone.0244062)
Supplement: S2 Table — (DOCX) [file pone.0244062.s006.docx]

| **Propensity score-matched** | | | |  |  |  |  |  |  |  |  |  |
| --- | --- | --- | --- | --- | --- | --- | --- | --- | --- | --- | --- | --- |
|  | **MACE** | | **Death** | | **Non-fatal MACE** | | **Revasc** | | **Shock** | | **Stroke** | |
|  | **HR (95% CI)** | **p-value** | **HR (95% CI)** | **p-value** | **HR (95% CI)** | **p-value** | **HR (95% CI)** | **p-value** | **HR (95% CI)** | **p-value** | **HR (95% CI)** | **p-value** |
| DES ,PDC ≥80% versus DES, PDC <80% | 0.364 (0.298 – 0.444) | < 0.001 | 0.216 (0.164 – 0.285) | < 0.001 | 0.216 (0.164 – 0.285) | < 0.001 | 1.005 (0.721 – 1.401) | 1.00 | 0.150 (0.062 – 0.364) | < 0.001 | 0.564 (0.373 – 0.854) | 0.007 |
| BMS, PDC ≥80% versus BMS, PDC <80% | 0.398 (0.332 -  0.478) | < 0.001 | 0.258 (0.203 – 0.328) | < 0.001 | 0.589 ( 0.464 – 0.748) | < 0.001 | 0.995 (0.717 –  1.381) | 1.00 | 0.331 (0.173 – 0.636) | < 0.001 | 0.388 (0.263 – 0.573) | < 0.001 |
| DES, PDC ≥80% versus BMS, PDC ≥80% | 0.754 (0.608 – 0.936) | 0.010 | 0.695 (0.506 – 0.954) | 0.023 | 0.768 (0.593 – 0.995) | 0.045 | 0.651 (0.467 – 0.907) | 0.011 | 0.426 (0.162 – 1.121) | 0.08 | 0.981 (0.638 – 1.508) | 0.09 |
| DES, PDC <80% versus BMS, PDC ≥80% | 2.106 (1.744 – 2.543) | < 0.001 | 3.273 (2.555 – 4.192) | < 0.001 | 1.367 (1.063 – 1.758) | 0.014 | 0.995 (0.714 – 1.387) | 0.98 | 2.875 (1.482 – 5.579) | 0.001 | 1.717 (1.126 – 2.618) | 0.011 |
| DES, PDC ≥80% versus BMS, PDC <80% | 0.303 (0.250 – 0.368) | < 0.001 | 0.183 (0.140 – 0.239) | < 0.001 | 0.446 (0.348 – 0.572) | < 0.001 | 0.643 (0.451 – 0.917) | 0.014 | 0.144 (0.060 – 0.347) | < 0.001 | 0.375 (0.256 – 0.548) | < 0.001 |
| DES, PDC <80% versus BMS, PDC <80% | 0.858 (0.731 – 1.006) | 0.06 | 0.868 (0.725 – 1.040) | 0.12 | 0.802 (0.631 – 1.020) | 0.07 | 0.768 (0.593 – 0.995) | 0.045 | 0.956 (0.568 – 1.608) | 0.87 | 0.675 (0.467 – 0.976) | 0.035 |

| **Propensity score-matched, landmark analysis** | | | | | | | |  |  |  |  |  |
| --- | --- | --- | --- | --- | --- | --- | --- | --- | --- | --- | --- | --- |
|  | **MACE** | | **Death** | | **Non-fatal MACE** | | **Revasc** | | **Shock** | | **Stroke** | |
|  | **HR (95% CI)** | **p-value** | **HR (95% CI)** | **p-value** | **HR (95% CI)** | **p-value** | **HR (95% CI)** | **p-value** | **HR (95% CI)** | **p-value** | **HR (95% CI)** | **p-value** |
| DES ,PDC ≥80% versus DES, PDC <80% | 0.610 (0.481 – 0.774) | < 0.001 | 0.443 (0.318 – 0.617) | < 0.001 | 0.666 (0.496 – 0.896) | 0.007 | 0.676 (0.454 – 1.008) | 0.05 | 0.281 (0.107 – 0.740) | 0.006 | 0.655 (0.412 – 1.040) | 0.07 |
| BMS, PDC ≥80% versus BMS, PDC <80% | 0.593 (0.476 – 0.739) | < 0.001 | 0.496 (0.374 – 0.657) | < 0.001 | 0.660 (0.498 – 0.874) | 0.003 | 1.238 (0.821 – 1.867) | 0.30 | 0.575 (0.248 – 1.331) | 0.20 | 0.338 (0.216 – 0.529) | < 0.001 |
| DES, PDC ≥80% versus BMS, PDC ≥80% | 0.795 (0.631 – 1.002) | 0.05 | 0.647 (0.465 – 0.900) | 0.009 | 0.846 (0.636 – 1.125) | 0.25 | 0.728 (0.502 – 1.054) | 0.09 | 0.536 (0.195 – 1.476) | 0.22 | 1.162 (0.717 – 1.884) | 0.54 |
| DES, PDC <80% versus BMS, PDC ≥80% | 1.299 (1.029 – 1.639) | 0.027 | 1.467 (1.083 – 1.987) | 0.013 | 1.258 (0.938 – 1.688) | 0.12 | 1.065 (0.727 – 1.559) | 0.75 | 1.911 (0.838 – 4.357) | 0.12 | 1.758 (1.071 – 2.885) | 0.024 |
| DES, PDC ≥80% versus BMS, PDC <80% | 0.468 (0.373 – 0.586) | < 0.001 | 0.323 (0.237 – 0.442) | < 0.001 | 0.549 (0.413 – 0.728) | < 0.001 | 0.892 (0.582 – 1.367) | 0.60 | 0.310 (0.116 – 0.826) | 0.013 | 0.388 (0.257 – 0.586) | < 0.001 |
| DES, PDC <80% versus BMS, PDC <80% | 0.770 ( 0.614 – 0.966) | 0.024 | 0.733 (0.553 – 0.973) | 0.031 | 0.821 (0.613 – 1.099) | 0.18 | 1.296 (0.838 – 2.005) | 0.024 | 1.097 (0.500 – 2.404) | 0.82 | 0.595 (0.388 – 0.911) | 0.016 |
