# Supplementary material for: Impact of medication adherence to dual antiplatelet therapy on the long-term outcome of drug-eluting or bare-metal stents
Source: PLoS One. 2020 Dec 16;15(12):e0244062. doi: 10.1371/journal.pone.0244062 (PMC7743933; doi:10.1371/journal.pone.0244062)
Supplement: S1 File — (DOCX) [file pone.0244062.s007.docx]

**S1 File. Administrative codes used for retrieve of clinical condition or procedure**

**RBC transfusion:**

NHIS (national health insurance service) code X2131-2, X2091-2, X2021-2, X2031-2, X211

**All-cause death:**

NIHS internal administrative code

**Revascularization:**

PCI using stent: NHIS M6561-4

PCI without stent: NHIS M6551-2 (balloon angioplasty), M6571-2 (atherectomy)

Bypass surgery: NHIS O1641-2,O1647, OA641-2, OA647.

**Shock: any of the following conditions:**

Resuscitation: NHIS M5873-7

Intubation and mechanical ventilation: NHIS M5850,M5857,M5858,M5860, M5859

Mechanical circulatory assistance device: NHIS O1921-2 (intra-aortic balloon counterpulsation), O1901-4 (extracorporeal membrane oxygenation)

**Stroke:**

Cerebral hemorrhage (ICD-10: I60-62), cerebral infarction (ICD-10: I63), or other stroke (ICD-10: I64, I67, I69) followed by admission and brain CT or MRI imaging within 7 days

**Administrative codes used for calculation of Charlson comorbidity index**

**Charlson comorbidity index assigns the following points for each of the following conditions:**

- - 6 points for AIDS (code:B20, B21, B22, B24) or metastatic solid tumor (code: C77, C78, C79, C80)
  - 3 points for moderate to severe liver disease (code: I850, I859, I864, I982,K704, K711, K721, K729, K765, K766, K767)
  - 2 points for any non-metastatic solid tumor, malignant lymphoma, leukemia (code: C00, C01, C02, … , C97), diabetes with end organ damage (code: E102, E103, E104, E105, E107, E112, E113, E114, E115, E117, E122, E123, E124,E125, E127, E132, E133, E134, E135, E137, E142, E143,E144, E145, E147), moderate to severe renal disease (code: N18, N19, I120, I131, N032, N033, N034, N035, N036, N037, N052, N053, N054, N055, N056,N057, N250, Z490, Z491, Z492, Z940, Z992), and hemiplegia (code: G81, G82, G041, G114, G801, G802, G830, G831, G832, G833, G834, G839)
  - 1 point for diabetes without end organ damage (code: E100, E101, E106, E108, E109, E110, E111, E116, E118, E119, E120, E121, E126, E128, E129, E130, E131, E136, E138, E139, E140, E141, E146, E148, E149), mild liver disease (code: B18, K73, K74, K700, K701, K702, K703, K709, K713, K714, K715, K717, K760, K762, K763, K764, K768, K769, Z944), ulcer disease (code: K25, K26, K27, K28), connective tissue disease (code: M05, M06, M32, M33, M34, M315, M351, M353, M360), chronic pulmonary disease (code: J40, J41, J42, J43, J44, J45, J46, J47, J60, J61, J62, J63, J64, J65, J66, J67, I278, I279, J684, J701, J703), dementia (code: F00, F01, F02, F03, G30, F051, G311), cerebrovascular disease (code: G45, G46, I60, I61, I62, I63, I64, I65, I66, I67, I68, I69, H340), peripheral vascular disease (code: I70, I71, I731, I738, I739, I771, I790, I792, K551, K558, K559, Z958, Z959), congestive heart failure (code: I43,I50, I099, I110, I130, I132,I255, I420, I425, I426, I427, I428, I429, P290), and myocardial infarction (code: I21, I22, I252).

The sum of points is used as a measure of 1-year mortality risk and burden of disease.
