# Supplementary material for: Impact of medication adherence to dual antiplatelet therapy on the long-term outcome of drug-eluting or bare-metal stents
Source: PLoS One. 2020 Dec 16;15(12):e0244062. doi: 10.1371/journal.pone.0244062 (PMC7743933; doi:10.1371/journal.pone.0244062)
Supplement: S2 File — (DOCX) [file pone.0244062.s008.docx]

**STROBE statement checklist of items (version 4) for the report of cohort study**

1. Title and abstract
   1. The study’s design was indicated with a commonly used term in both the title and the abstract
      1. In the title:
         - ‘Impact of medication adherence to dual antiplatelet therapy on the long-term outcome of drug-eluting or bare-metal stents
      2. In the abstract:
         - ‘We retrospectively enrolled all Koreans undergoing PCI with either DES or BMS in year 2011 (n=47,291) and investigated 5-year clinical outcome after reflecting the clinical propensity for receiving DES or BMS and proportion of days covered (PDC) of DAPT. Primary outcome was the 5-year major adverse clinical event (MACE) rate consisting all-cause death, revascularization, shock, or stroke. Good medication adherence was defined by PDC ≥80% in the first 6 month.’
   2. Provide in the abstract an informative and balanced summary of what was done and what was found
      1. What was done:
         - ‘In this real-world data study, DES is superior to BMS in terms of efficacy and safety even after reflecting underlying clinical profiles and medication adherence to DAPT.’
2. Introduction, background/rationale
   1. Scientific background and rationale for investigation
      1. Page 4, introduction:
         - ‘Currently, limited data is available for the safety or efficacy of DES compared with BMS with respect to the medication adherence to DAPT.’
3. Introduction, objective
   1. Study-specific objective
      1. Page 5, introduction:
         - ‘We retrospectively investigated the outcome of patients who underwent PCI with DES or BMS with respect to the medication adherence to DAPT using a real-world data.’
4. Methods, study design
   1. Presenting key elements of study design early in the paper
      1. Shown in page 5 – 6 and also in Figure 1
5. Methods, setting
   1. The setting
      1. Methods, Page 5
         - ‘This study is retrospective cohort study using public administrative database’
   2. Location
      1. Methods, Page 5
         - ‘the National Healthcare Insurance Service of Korea’
   3. Relevant dates including periods of recruitment, exposure, follow-up
      1. Methods, Page 5 – 6
         - ‘All procedures and clinical events were defined with the timestamp of claims. The index PCI date was the date of first PCI performed in the selection period from January 1, 2011 to December 31, 2011. Baseline clinical characteristics or medical history prior to the index PCI were defined by respective ICD-10 codes or claims issued in the look-back period from January 1, 2009 to one day before the index PCI date. The procedure or clinical event after PCI were defined by the issued claims and ICD-10 codes in the follow-up period from the index PCI date to the end of study period’
   4. Data collection
      1. Methods, Page 5,
         - ‘Data was retrieved retrospectively on July 1, 2017’
6. Methods, participants
   1. Eligibility criteria and the sources and methods of selection of participants
      1. Methods, Page 5
         - ‘The study cohort consisted of anonymized individual claims of PCI using stent from January 1, 2011 to December 31, 2011’
   2. The methods of follow-up
      1. Methods, Page 5
         - ‘Administrative claims, medical services claims, pharmacy claims, and death records issued for these patients from January 1, 2009 through December 31, 2016 were retrieved to assess pre-PCI clinical status and clinical events in the post-PCI follow-up period’
7. Methods, variables
   1. Definition of all outcomes, exposures, predictors, potential confounders, and effect modifiers
      1. Outcome: Methods, Page 5
         - ‘Administrative claims, medical services claims, pharmacy claims, and death records issued for these patients from January 1, 2009 through December 31, 2016 were retrieved to assess pre-PCI clinical status and clinical events in the post-PCI follow-up period’
      2. Exposures, predictors, potential confounders and effect modifiers:
         - Not applicable
   2. Definition of diagnostic criteria
      1. Methods, Page 5
         - The study cohort consisted of anonymized individual claims of PCI using stent from January 1, 2011 to December 31, 2011
8. Methods, data sources/measurement
   1. For each variable of interest, give sources of data and details of methods of assessment (measurement). Describe comparability of assessment methods if there is more than one group
      1. Methods, page 6, clinical events:
         - Administrative data with timestamp
      2. Statistical analysis, page 7, comparability of assessment:
         - ‘Comparison of cumulative events in DES and BMS groups are compared using propensity-score matched survival analysis and Cox proportional hazard model’
9. Methods, bias
   1. Any efforts to address potential sources of bias
      1. Statistical analysis, page 7:
         - For survival analyses, the potential confounding factors in the use of DES or BMS were adjusted by matching patient’s propensity for DES or BMS.
10. Methods, study size
    1. Explain how the study size was arrived at
       1. Methods, page 5:
          - ‘The study cohort consisted of anonymized individual claims of PCI using stent from January 1, 2011 to December 31, 2011’
11. Methods, quantitative variables
    1. Statistical methods
       1. Propensity score matching: to balance patient’s propensity for transfusion.
       2. Cox proportional hazard model: for survival analysis
    2. Methods to examine subgroups and interactions
       1. Statistical analysis, page 7 – 8:
          - ‘The impact of transfusion on clinical subgroups including age, gender, clinical risk factors and comorbidities, prior history of cardiovascular events, prior history of transfusion, initial angina or myocardial infarction as clinical presentation, and number of stents, periprocedural transfusion, Charlson’s comorbidity index, and DAPT PDC of first 6 month were assessed using hazard ratio. Interaction in each clinical subgroup was also assessed’
    3. How missing data and lost follow-up were addressed
       1. Methods, page 6
          - ‘No patient was lost to follow-up with respect to death. Follow-up of non-fatal clinical event was completed for 99.2% of the 4th year and 94.8% of the 5th year entries’
12. Results, participants
    1. The number of individuals at each stage of study
       1. Shown in table and N at risk in the each survival curve.
    2. Non-participation
       1. Not applicable because this study is retrospective cohort study using administrative database.
    3. Flow diagram
       1. Shown in Figure 1.
13. Results, descriptive data
    1. Characteristics of study participants and information on exposures and potential confounders
       1. Clinical characteristics are shown in Table 1.
    2. Number of participants with missing data for each variable of interest
       1. No patient was lost to follow-up with respect to death.
       2. Follow-up of non-fatal clinical event was available for 99.2% at 4 years and 94.8% at 5 years.
14. Results, outcome data
    1. Reports of numbers of outcome events or summary measures over time.
       1. Shown in each figures in Figure 2 – 5.
15. Results, main results
    1. Unadjusted estimates and confounder-adjusted estimates with 95% confidence interval.
       1. Unadjusted survival analysis in Figure 2
       2. Propensity-score matched survival analysis in Figure 3
       3. Landmark analysis of propensity-score matched survival in Figure 5
    2. Category boundaries used in categorization of continuous variable
       1. Good medication adherence was define by
          - PDC of DAPT ≥ 80% or < 80%.
    3. Translating estimates of relative risk into absolute risk for a meaningful time period
       1. Results, page 9 – 10,
          - Unadjusted analysis: In the analysis of whole cohort, patients that received DES had much lower 5-year cumulative risk of MACE compared to patients that received BMS (37.8% versus 56.3%, HR = 0.574 [95% CI = 0.526 – 0.628], p < 0.001).
          - Adjusted analysis: In the survival analysis of propensity matched 934 pairs, the 5-year cumulative risk of MACE of patients that received DES was lower than patients that received BMS (45.9% versus 54.4%, HR = 0.796, 95% CI = 0.700 – 0.905, p < 0.001).
16. Results, other analyses
    1. Analysis of subgroups and interactions, or sensitivity analysis
       1. Subgroup analyses with interaction are shown in Supplementary Figure II.
17. Discussion, key results
    1. Summarize key results with reference to study objectives
       1. Conclusion,
          - In this real-world cohort data of patients undergoing PCI, the clinical outcome of DES was better than BMS even after reflecting clinical risk and medication adherence to DAPT.
18. Discussion, Limitations
    1. Limitations, sources of potential bias or imprecision.
       1. Details are described in the final paragraph of Discussion section.
19. Discussion, Interpretation
    1. Cautious overall interpretation of results considering objectives, limitations, multiplicity of analyses, results from similar studies, and other relevant evidences
       1. Discussion section:
          - ‘In this retrospective analysis of a nationwide all-comer real-world data, DES was better than BMS in the composite outcome of death and non-fatal hard clinical events including revascularization, critically ill cardiovascular status, and stroke for up to 5 years. This finding was consistent even after reflecting baseline clinical profiles and the first 6-month medication adherence to DAPT’
       2. Discussion, page 11- 12, results from similar studies:
          - ‘Three large meta-analyses showed that not only the risk of revascularization but also the risks of stent thrombosis and myocardial infarction were lower in DES compared to BMS. In these meta-analyses, medication adherence to DAPT was not analyzed.
          - However, NORSTENT trial, the largest of randomized trials that compared DES with BMS, did not show difference in the composite outcome of all-cause death and nonfatal myocardial infarction between DES and BMS.NORSTENT enrolled 44% of Norwegian PCI in the study period, and excluded potentially high risk patients with prior revascularization, 2-stent technique for bifurcation, or life expectancy of less than 5 years. DAPT was prescribed for 9-month regardless of stent type. Our study showed numerically similar long-term revascularization rate (16.7% at 5 year versus 15.9% at 6 year) and much higher all-cause death rate (18.6% at 5 year versus 7.0% at 6 year) compared to NORSTENT. Unlike NORSTENT, our all-comer study did not excluded any PCI using stent. DAPT was much less frequently prescribed in patients that received BMS. Enrollment of all patients including high risk profile patients might be translated into lower clinical efficacy of BMS compared to DES’
20. Discussion, generalisability
    1. Generalisability or external validation
       1. The use of nationwide all-comer cohort without any specific inclusion or exclusion parameters may increase the generalisability of our results.
       2. The use of parameters consisting of administrative database without laboratory results or medical records is a major limitation of this study, but may be easily validated with the other administrative database.
21. Funding
    1. Funding sources are shown in the Acknowledgement section.
